# Group testing can improve the cost-efficiency of prospective-retrospective biomarker studies

**DOI:** 10.1186/s12874-021-01239-4

**Published:** 2021-03-19

**Authors:** Wei Zhang, Zhiwei Zhang, Julia Krushkal, Aiyi Liu

**Affiliations:** 1 LSC, Academy of Mathematics and Systems Science, Chinese Academy of Sciences, Beijing, China; 2 Biometric Research Program, Division of Cancer Treatment and Diagnostics, National Cancer Institute, National Institutes of Health, Bethesda, MD 20892 USA; 3 Biostatistics and Bioinformatics Branch, Eunice Kennedy Shriver National Institute of Child Health and Human Development, National Institutes of Health, Bethesda, MD USA

**Keywords:** Biomarker study design, Cost-efficiency, Group testing, Pooling, Two-phase sampling

## Abstract

**Background:**

Cancer treatment is increasingly dependent on biomarkers for prognostication and treatment selection. Potential biomarkers are frequently evaluated in prospective-retrospective studies in which biomarkers are measured retrospectively on archived specimens after completion of prospective clinical trials. In light of the high costs of some assays, random sampling designs have been proposed that measure biomarkers for a random sub-sample of subjects selected on the basis of observed outcome and possibly other variables. Compared with a standard design that measures biomarkers on all subjects, a random sampling design can be cost-efficient in the sense of reducing the cost of the study substantially while achieving a reasonable level of precision.

**Methods:**

For a biomarker that indicates the presence of some molecular alteration (e.g., mutation in a gene), we explore the use of a group testing strategy, which involves physically pooling specimens across subjects and assaying pooled samples for the presence of the molecular alteration of interest, for further improvement in cost-efficiency beyond random sampling. We propose simple and general approaches to estimating the prognostic and predictive values of biomarkers with group testing, and conduct simulation studies to validate the proposed estimation procedures and to assess the cost-efficiency of the group testing design in comparison to the standard and random sampling designs.

**Results:**

Simulation results show that the proposed estimation procedures perform well in realistic settings and that a group testing design can have considerably higher cost-efficiency than a random sampling design.

**Conclusions:**

Group testing can be used to improve the cost-efficiency of biomarker studies.

**Supplementary Information:**

The online version contains supplementary material available at 10.1186/s12874-021-01239-4.

## Background

### Biomarkers and biomarker studies

A biomarker is “a characteristic that is measured as an indicator of normal biological processes, pathogenic processes, or responses to an exposure or intervention” [1]. Biomarkers play increasingly important roles in the treatment of cancer and other disease conditions [2–4]. A biomarker is said to be prognostic if it is associated with clinical outcomes in the absence of therapy or in the setting of some therapy that most patients are likely to receive (e.g., standard of care). A biomarker is said to be predictive if it is related to the effect of one treatment versus another. A predictive biomarker must be prognostic for at least one of the two treatments being compared. On the other hand, a prognostic biomarker does not need to be predictive. Both types of biomarker are of great interest in contemporary clinical research and practice.

The prognostic or predictive value of a biomarker can be evaluated in a variety of study settings with varying levels of evidence [5]. The highest level of evidence is attained by a fully prospective clinical study in which patients are prospectively enrolled, treated, and followed for clinical outcomes, with specimens collected at baseline and assayed in real time for marker values. Such a study can be highly expensive and may take many years to complete. By the time the study is completed, the biomarker may have become obsolete. A practical alternative to this fully prospective approach is a two-phase prospective-retrospective (P-R) clinical study which differs from a fully prospective study in that baseline specimens are archived after collection and assayed later for specific biomarkers [5]. This P-R approach can save a great deal of time for biomarker researchers by allowing them to focus their efforts on assaying archived specimens from completed clinical trials. This approach has been used successfully to validate KRAS as a predictive biomarker in colorectal cancer [6, 7] and is now commonly adopted for biomarker studies [5, 8].

P-R studies are time-efficient but can be rather costly due to the high costs of some molecular assays such as next generation sequencing [9]. To improve the cost-efficiency of P-R studies, random sampling (RS) designs have been proposed that measure biomarkers for a random sub-sample of subjects selected on the basis of observed outcome and possibly other variables. Examples of RS designs include the case-cohort and nested case-control designs [10, 11]. If the outcome of interest is an infrequent event, it is generally advisable to over-sample cases (i.e., subjects who had the event) for biomarker measurement. The RS design has the potential to be cost-efficient in the sense of attaining a higher level of precision on a per-assay basis than the standard design (for example, using 50% of the assays to produce 60% of the precision as compared to the standard design). On the other hand, it does not make use of all available specimens, raising questions about the possibility of further improvement.

### Group testing

In this article, we explore the use of group testing (GT) to further improve the cost-efficiency of P-R studies (beyond the RS design) when the biomarker of interest indicates the presence of some molecular alteration (e.g., mutation in a gene). GT refers to the practice of physically pooling specimens across subjects and assaying pooled samples for the presence of the molecular alteration in the pool. For an assay with negligible error, a positive test result for a pooled sample would indicate that the molecular alteration is present in one or more subjects in the pool, while a negative test result would indicate the contrary. Since its introduction by Dorfman [12] as a cost-efficient way of screening for syphilis, GT has been applied to many different areas of biomedical research including virology [13–15], genetics [16–19], drug development [20], and most recently Covid-19 [21–23]. In particular, the feasibility and performance of GT for detecting mutations in tumor have been investigated with promising results [17–19]. Possible motivations for GT include cost-efficiency, statistical efficiency [24, 25], limited availability of specimens, and confidentiality concerns [26]. Some authors have considered the use of GT in retrospective epidemiologic studies [25, 27], but the potential utility of GT in P-R biomarker studies seems largely unnoticed.

This commentary provides a statistical investigation of the potential utility of GT to improve the cost-efficiency of P-R biomarker studies beyond that achieved by the RS design. Efficiency comparisons will be made with or without adjusting for the number of assays required. We will consider a simple yet common situation with a dichotomous outcome, where GT is performed on a dichotomous biomarker in an outcome-dependent fashion, under the assumption that assay error is negligible. We extend the methods in References [25, 27] to this situation and develop simple procedures for estimating the prognostic or predictive value of a biomarker measured by GT. The main ideas are described in the text with technical details provided in an online supplement. Simulation studies are conducted to evaluate the proposed estimation procedures as well as the statistical efficiency and cost-efficiency of the GT design in comparison to the standard design and the RS design.

### Study setting

**Fig. 1:**
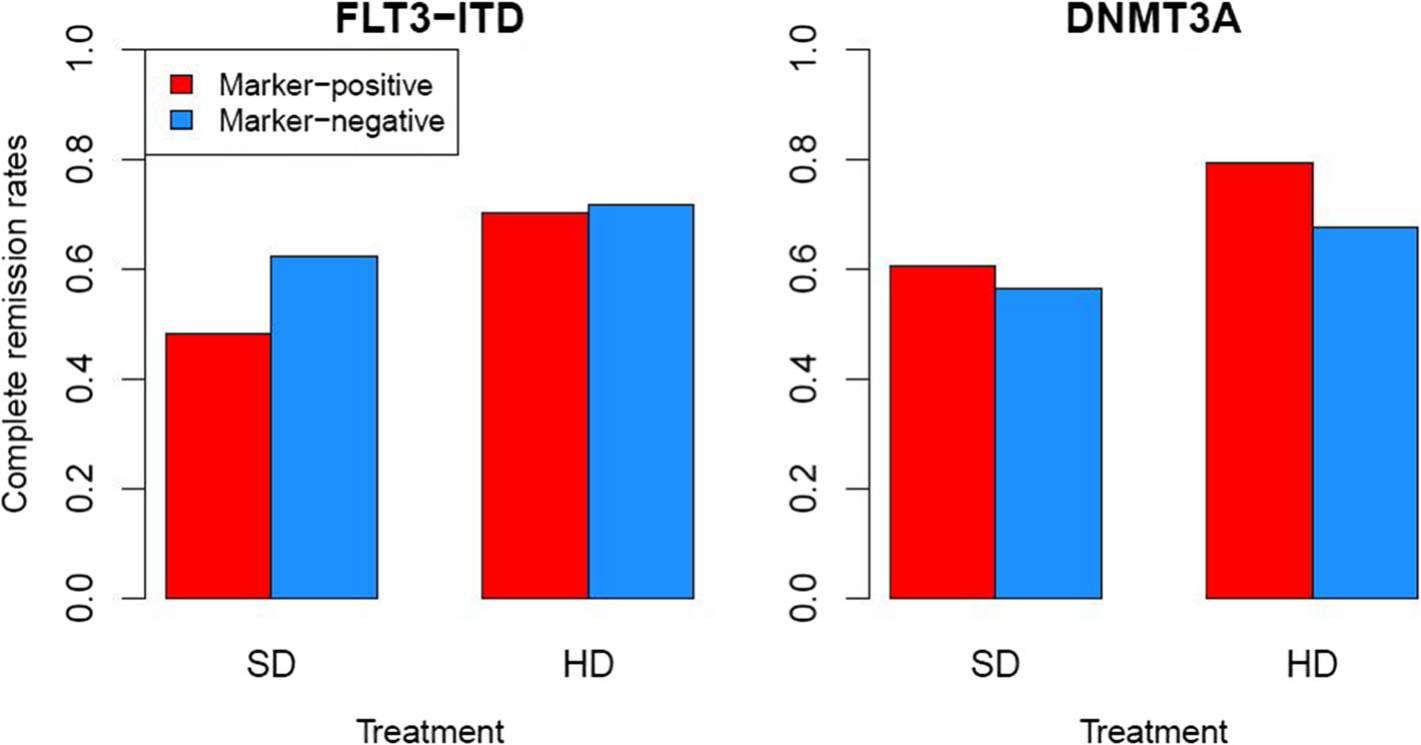
Observed complete remission rates by treatment and marker status for two biomarkers (FLT3-ITD and DNMT3A) in the E1900 trial

The ideas will be illustrated using the ECOG-ACRIN Cancer Research Group trial E1900 (NCT00049517), a randomized clinical trial comparing high-dose (HD) daunorubicin (90 mg/m^2^) with standard-dose (SD) daunorubicin (45 mg/m^2^) for patients 17–60 years of age with de novo untreated acute myeloid leukemia [28]. A total of 657 patients were randomized in a 1:1 ratio and followed for a median of 80.1 months. The trial demonstrated significant benefits of HD versus SD with respect to overall survival (hazard ratio 0.74; 95% CI 0.61–0.89; *P* = 0.001) and complete remission (odds ratio 1.79; 95% CI 1.27–2.52; *P* = 0.001). For illustration, we will use complete remission as the dichotomous outcome of interest, even though it was not the primary outcome of the trial. The trial had several biomarkers of interest, including the FLT3-ITD internal tandem duplication variant and mutation in DNMT3A, both of which were present in 24% of the trial participants. Figure 1 shows the observed complete remission rates for HD and SD in each biomarker sub-group. Both biomarkers were assayed using PCR amplification and bidirectional Sanger sequencing [29]. Because such assays have near-perfect sensitivity and specificity [30], we will focus on perfect assays in the main text and present estimation methods and simulation results for less-than-perfect assays in the online supplement.

## Methods

### Evaluating a prognostic biomarker

Evaluation of a prognostic biomarker, say *X*, usually focuses on its association with an outcome variable, say *Y*, for a given treatment, which is fixed in this section and therefore suppressed from the notation. We assume that *X* is a binary indicator of some molecular alteration (e.g., mutation); so *X* = 1 if the alteration is present and 0 otherwise. A patient is said to be “marker-positive” if *X* = 1 and “marker-negative” if *X* = 0. For simplicity, we assume here that *Y* is also binary (0 or 1) with *Y* = 1 representing treatment response (e.g., complete remission). A patient with *Y* = 1 is said to be a responder. In this setting, the association between *X* and *Y* may be assessed by comparing the marker-specific response rates *p*_1_ and *p*_0_, where *p*_*x*_ = *P*(*Y* = 1| *X* = *x*), *x* = 0, 1. Common measures of association include the log-odds ratio *log*[*p*_1_(1 − *p*_0_)/{*p*_0_(1 − *p*_1_)}], the log-ratio *log*(*p*_1_/*p*_0_), and the difference *p*_1_ − *p*_0_ [31, 32]. Each of these can be written as *g*(*p*_1_) − *g*(*p*_0_), where *g* is, respectively, the logit function, the log function, or the identity function.

**Fig. 2:**
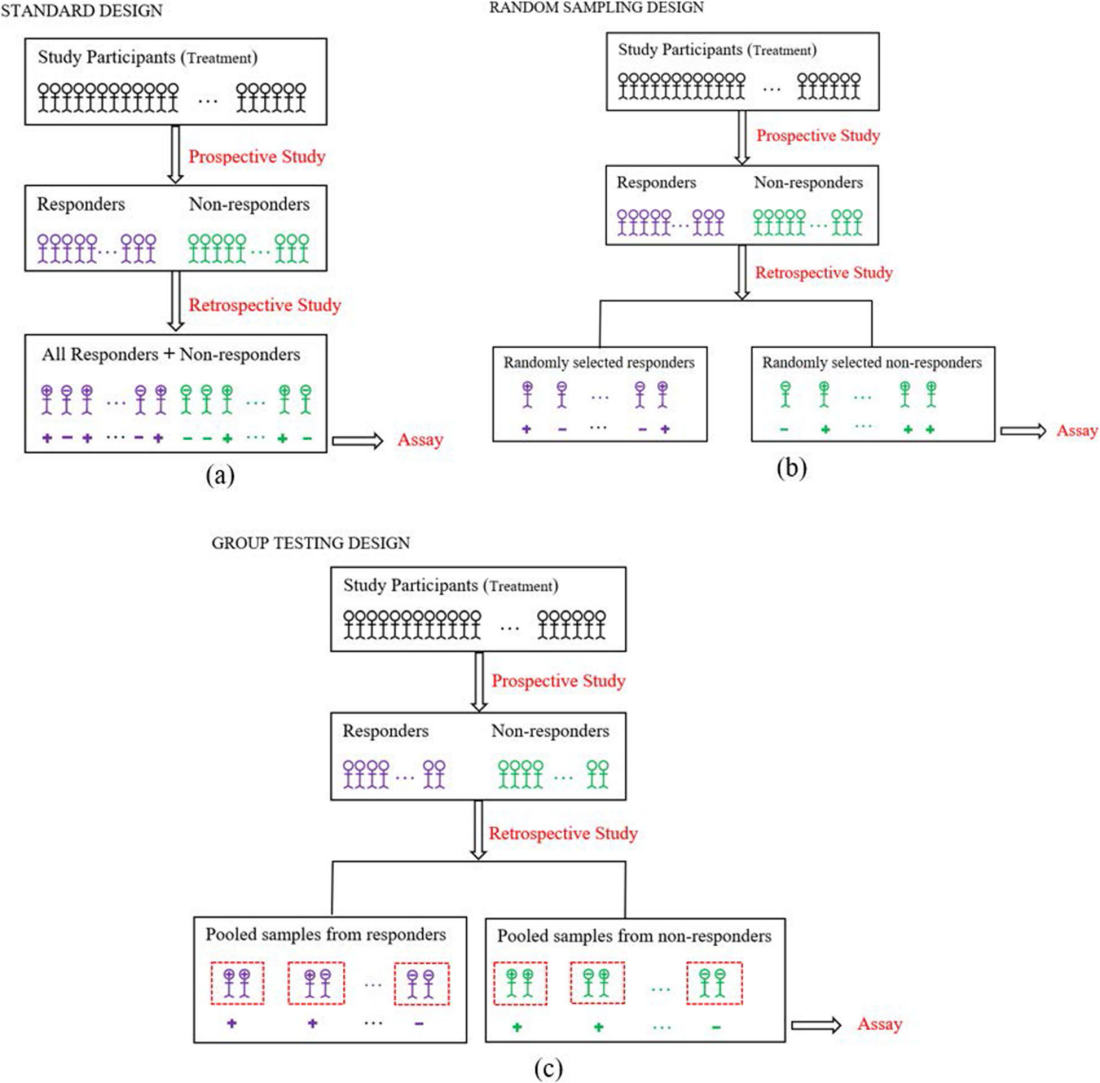
Schematics for the standard (**a**), random sampling (**b**) and group testing (**c**) designs for evaluating a prognostic biomarker

Suppose a clinical trial has been completed to yield outcome data for a random sample of *n* subjects (either in a one-arm trial or in one arm of a multi-arm trial), together with archived specimens available for biomarker studies. As shown in Fig. 2a, a standard P-R study of the biomarker *X* would entail assaying all specimens of individual subjects and measuring the biomarker for each individual subject. From such data it is straightforward to estimate *p*_1_ (*p*_0_) as the proportion of responders among the marker-positive (negative) subjects, which can then be substituted into any measure of association. For illustration, the upper portion of Table 1 shows point estimates and standard errors of the three association measures mentioned earlier for the two biomarkers (FLT3-ITD and DNMT3A) in the two treatment groups (HD and SD) of the E1900 trial.

**Table 1:** Point estimates (standard errors) of various measures of association and interaction in the E1900 trial

Parameter Type	Treatment	Measure/link	FLD3-ITD	DNMT3A
Association	HD	log-OR	−0.06 (0.31)	0.63 (0.33)
		log-ratio	−0.02 (0.09)	0.16 (0.08)
		difference	−0.02 (0.06)	0.12 (0.06)
	SD	log-OR	−0.58 (0.25)	0.18 (0.26)
		log-ratio	−0.26 (0.12)	0.07 (0.11)
		difference	−0.14 (0.06)	0.04 (0.06)
Interaction		logit	0.52 (0.39)	0.46 (0.41)
		log	0.24 (0.15)	0.09 (0.13)
		identity	0.13 (0.09)	0.08 (0.08)

*OR* Stands for odds ratio

Under the RS design, subjects are selected randomly, typically in an outcome-dependent manner, for measurement of *X*, as illustrated in Fig. 2b. Let *n*_1_ (*n*_0_) denote the total number of responders (non-responders) in the trial, and let *m*_1_ (*m*_0_) denote the number of responders (non-responders) to be selected for measurement of *X*. If treatment response is rare (i.e., *n*_1_ is very small), it is common to select all responders (i.e., *m*_1_ = *n*_1_) and a comparable number of non-responders. Similar considerations apply to the opposite situation where treatment non-response is rare and *n*_0_ is very small. The RS design permits direct estimation of the prevalence of marker-positives among responders and non-responders, formally defined as the conditional probabilities *q*_*y*_ = *P*(*X* = 1| *Y* = *y*), *y* = 0, 1. Specifically, *q*_1_ (*q*_0_) is estimated by the proportion of marker-positives among the *m*_1_ responders (*m*_0_ non-responders) selected for biomarker measurement. These estimates alone are sufficient for estimating the odds ratio for *X* and *Y*. For other measures of association, Bayes’ theorem can be used to obtain estimates of *p*_1_ and *p*_0_, which can then be substituted into any measure of association. These and other technical details are provided in the online supplement.

The GT design is a generalization of the RS design which allows more subjects to be assayed, though not necessarily on an individual basis. Figure 2c gives an example GT design for the same P-R study with the same numbers of assays for responders (*m*_1_) and non-responders (*m*_0_) as required by the RS design in Fig. 2b. Compared to the RS design, the GT design allows assaying twice as many responders and non-responders with the potential to produce more information. In general, the GT design is a stratified (by outcome) pooling design, and the pool sizes (i.e., number of subjects in a pool) for responders and non-responders may or may not be the same. If the pool size is equal to 1 in both strata, the GT design reduces to the RS design. In each stratum of the GT design, the marker prevalence *q*_*y*_ can be estimated with pooled assay data using a maximum likelihood approach [20]. These estimates can be used in the same manner as in the RS design to estimate any measure of association between *X* and *Y*.

These designs are compared in a simulation study mimicking the E1900 trial. A separate simulation experiment is conducted for each combination of treatment group (HD or SD) and biomarker (FLT3-ITD or DNMT3A). Each experiment consists of 10,000 replicate trials in which *T* is fixed, *X* is generated randomly with *P*(*X* = 1) ≈ 0.24 (observed proportion), *Y* is generated conditionally on (*T*, *X*) according to the observed proportions in Fig. 1, and the sample size is the same as the actual size of the treatment group (327 for HD; 330 for SD). Each simulated trial is used to assess the prognostic value of *X* under the standard, RS and GT designs. The RS design is implemented in two versions which assay approximately one half (RS-2) or one third (RS-3) of the trial participants and which attempt to assay equal numbers of responders and non-responders to the extent possible. Accordingly, the GT design is also implemented in two versions which match the RS designs in the number of assays and which attempt to use a group size of 2 (GT-2) or 3 (GT-3) to the extent possible.

### Evaluating a predictive biomarker

We now consider the problem of evaluating a predictive biomarker for choosing between an experimental treatment (*T* = 1) and a standard treatment (*T* = 0) in a randomized clinical trial. Let *X* and *Y* be defined as in the last section and note that *T* is independent of *X* by randomization. The predictive value of *X* can be quantified by the *T*- *X* interaction in a regression model relating *Y* to (*T*, *X*) . For a binary *Y*, such a regression model may be specified as

1 $$ g\left\{P\left(Y=1|T,X\right)\right\}={\upbeta}_1+{\upbeta}_TT+{\upbeta}_XX+{\upbeta}_{TX} TX, $$

where *g* is a specified link function which is commonly chosen to be the logit, log or identity function. For any link function, the interaction coefficient β_*TX*_ can be interpreted as a “difference in difference”:

2 $$ {\upbeta}_{\mathrm{TX}}=\left\{g\left({P}_{11}\right)-g\left({P}_{10}\right)\right\}-\left\{g\left({P}_{01}\right)-g\left({P}_{00}\right)\right\}, $$

where *p*_*tx*_ = *P*(*Y* = 1| *T* = *t*, *X* = *x*), *t*, *x* = 0, 1.

Suppose a randomized clinical trial has been completed to produce treatment and outcome data on a random sample of *n* subjects, together with archived specimens available for measurement of *X*. A standard P-R biomarker study would simply measure *X* for each individual subject in the trial, which requires a total of *n* assays. The resulting data can be used to fit model (1) and estimate β_*TX*_ using standard software. Alternatively, one can estimate each *p*_*tx*_ as the proportion of responders among subjects in the *T* = *t* treatment group with marker status *X* = *x*, and substitute these estimates into Eq. (2) to estimate β_*TX*_. These two approaches are generally equivalent. The lower portion of Table 1 shows the results (point estimates and standard errors) of estimating β_*TX*_ for the aforementioned three link functions in the E1900 trial.

The RS design involves random selection of subjects for measurement of *X*, which may be stratified on treatment and outcome; this can be illustrated with two copies of Fig. 2b, one for each treatment group. Let *n*_*ty*_ denote the total number of subjects available in the (*T* = *t*, *Y* = *y*) stratum, and let *m*_*ty*_ denote the number of subjects to be selected for measurement of *X* in the same stratum. Conventional wisdom suggests that the *m*_*ty*_ ’s should be made comparable to each other, which may require over-sampling subjects in small strata. The RS design permits direct estimation of the prevalence of marker-positives in each treatment-outcome stratum, formally defined as the conditional probabilities *q*_*ty*_ = *P*(*X* = 1| *T* = *t*, *Y* = *y*), *t*, *y* = 0, 1. Specifically, each *q*_*ty*_ is estimated by the proportion of marker-positives among the *m*_*ty*_ subjects in the (*T* = *t*, *Y* = *y*) stratum who are selected for biomarker measurement. For the logit link, these estimates suffice for estimating β_*TX*_. For other link functions, Bayes’ theorem can be used to combine these estimates of *q*_*ty*_ ’s with the fully observed treatment and outcome data to estimate all *p*_*tx*_ ’s and hence β_*TX*_.

A GT design in this context is essentially a stratified (by treatment and outcome) pooling design and can be thought of as two copies of Fig. 2c, one for each treatment group. Compared with an RS design with the same number of assays (*m*_*ty*_) in each treatment-outcome stratum, a GT design with pool size 2 allows twice as many subjects to be assayed (though not on an individual basis) in an attempt to produce more information. In general, a GT design may prescribe pooling in some or all treatment-outcome strata, and the pool size may or may not vary across strata. The RS design can be seen as a special type of GT design in which the pool size is equal to 1 in each stratum. In each treatment-outcome stratum of a general GT design, the marker prevalence *q*_*ty*_ can be estimated with pooled assay data using a maximum likelihood approach [20]. These estimates can be used in the same manner as in the RS design to estimate β_*TX*_ for any link function.

These designs are compared via simulation in the setting of the E1900 trial, with a separate simulation experiment for each biomarker (FLT3-ITD or DNMT3A). Each experiment consists of 10,000 replicate trials in which *T* and *X* are independently generated with *P*(*T* = 1) = 0.5 and *P*(*X* = 1) ≈ 0.24, *Y* is generated conditionally on (*T*, *X*) according to the observed proportions in Fig. 1, and the sample size is the same as the actual size of the trial (657). Each simulated trial is used to assess the predictive value of *X* under the standard, RS and GT designs. The RS design is implemented in two versions which assay approximately one half (RS-2) or one third (RS-3) of the trial participants and which attempt to perform the same number of assays in each stratum defined by (*T*, *Y*). Accordingly, the GT design is also implemented in two versions which match the RS designs in the number of assays and which attempt to use a group size of 2 (GT-2) or 3 (GT-3) in each stratum.

### Measures of performance

The performance of various designs is assessed in terms of relative efficiency and relative cost-efficiency, both of which are relative to the standard design, for estimating the association/interaction measure of interest. The relative efficiency of a non-standard design is defined as the ratio of the estimation variance for the standard design to that for the non-standard design in question. A GT-2 design with a relative efficiency of 0.85, for example, retains 85% of the information (i.e., precision) with half of the assays required by the standard design. The relative cost-efficiency of a non-standard design is defined as its relative efficiency multiplied by the ratio of the number of assays for the standard design to that for the non-standard design in question. For example, a GT-3 design with a relative cost-efficiency of 2 yields twice as much information as does the standard design on a per-assay basis.

### Choosing a pool size

Implementing the GT design requires choosing a pool size for each pooling stratum (based on outcome and possibly treatment). While we do not attempt to answer this question in full in this article, we provide some statistical insights here on how to choose a pool size to maximize cost-efficiency. As we explain in the online supplement, the statistical efficiency for estimating an association/interaction measure depends on the amount of available information (known in statistics as Fisher information) about the prevalence of the biomarker in each pooling stratum. Assuming that a fixed number of assays has been allocated to a given stratum with sufficient subjects/samples for all realistic pool sizes, the question then becomes how to choose a pool size to maximize the Fisher information about marker prevalence in a single pooled assay result. This per-assay Fisher information can be calculated analytically as a function of the true prevalence for each candidate pool size. This information, together with a preliminary estimate of the stratum-specific marker prevalence, provides a starting point for choosing a stratum-specific pool size, which can then be validated or revised on the basis of other considerations such as number of subjects, sample availability, pooling feasibility, and assay performance.

## Results

### Evaluating a prognostic biomarker

Simulation results for evaluating a prognostic biomarker are shown in Table 2. As expected, all five designs yield nearly unbiased estimates of association measures (results not shown). For the RS and GT designs, Table 2 presents simulation results of relative efficiency and relative cost-efficiency. The RS and GT designs are expected to have relative efficiency less than 1 because they use fewer assays than the standard design. Comparing RS and GT designs with the same number of assays, the GT design is clearly and substantially more efficient than the RS design. For studying DNMT3A in the SD group, the GT-3 design achieves 70–71% of the precision level of the standard design while requiring only one third of the assays, and is more than twice as efficient as the RS-3 design with the same number of assays. The other scenarios follow the same pattern with slightly different numbers. In Table 2, the relative cost-efficiency ranges between 0.94 and 1.27 for the RS designs, indicating that the RS designs are either similar or superior to the standard design in cost-efficiency. It is worth noting that the GT designs attain much higher levels of relative cost-efficiency (1.65–1.79 for GT-2; 1.94–2.40 for GT-3). In summary, the results in Table 2 indicate that RS and GT designs are usually cost-efficient as compared to the standard design, and that GT designs can achieve much higher cost-efficiency than RS designs.

**Table 2:** Simulation results for evaluating a prognostic biomarker in the setting of the E1900 trial

Biomarker	Treatment	Measure of Association	Relative Efficiency				Relative Cost-Efficiency			
			RS-2	RS-3	GT-2	GT-3	RS-2	RS-3	GT-2	GT-3
FLD3-ITD	HD	log-OR	0.61	0.38	0.87	0.76	1.22	1.13	1.75	2.29
		log-ratio	0.57	0.34	0.87	0.76	1.15	1.03	1.74	2.26
		difference	0.60	0.37	0.87	0.76	1.19	1.12	1.75	2.29
	SD	log-OR	0.50	0.33	0.82	0.65	1.01	0.98	1.65	1.94
		log-ratio	0.49	0.31	0.83	0.66	0.98	0.94	1.65	1.97
		difference	0.51	0.34	0.83	0.65	1.02	1.01	1.65	1.96
DNMT3A	HD	log-OR	0.64	0.41	0.89	0.80	1.27	1.23	1.79	2.40
		log-ratio	0.60	0.39	0.89	0.78	1.20	1.17	1.78	2.33
		difference	0.61	0.41	0.89	0.78	1.23	1.22	1.78	2.35
	SD	log-OR	0.50	0.33	0.85	0.71	1.00	1.00	1.70	2.13
		log-ratio	0.50	0.33	0.85	0.70	0.99	0.99	1.70	2.11
		difference	0.51	0.34	0.85	0.71	1.01	1.03	1.71	2.13

### Evaluating a predictive biomarker

Simulation results for evaluating a predictive biomarker are shown in Table 3. As in the case of evaluating a prognostic biomarker, estimation bias is negligible for each interaction measure in each design (results not shown). Therefore, our comparison of designs is focused on (cost-)efficiency. For the RS and GT designs, Table 3 presents simulation results of relative efficiency and relative cost-efficiency. In this setting, the RS designs are largely similar in cost-efficiency to the standard design, with relative cost-efficiency ranging from 0.89 to 1.13. In contrast, the GT designs are highly competitive in terms of relative cost-efficiency (1.72–1.75 for GT-2; 2.10–2.25 for GT-3). Thus, the simulation results in Table 3 demonstrate that GT designs are much more cost-efficient than the standard and RS designs for estimating an interaction measure. This can be an important advantage when the cost of a biomarker study is driven by the cost of assays.

**Table 3:** Simulation results for evaluating a predictive biomarker in the setting of the E1900 trial

Biomarker	Link for	Relative Efficiency				Relative Cost-Efficiency			
	Interaction	RS-2	RS-3	GT-2	GT-3	RS-2	RS-3	GT-2	GT-3
FLD3-ITD	logit	0.54	0.34	0.86	0.70	1.08	1.01	1.72	2.10
	log	0.50	0.30	0.86	0.71	0.99	0.89	1.72	2.13
	identity	0.53	0.34	0.86	0.70	1.06	1.01	1.72	2.11
DNMT3A	logit	0.56	0.35	0.88	0.75	1.13	1.05	1.75	2.25
	log	0.52	0.32	0.86	0.73	1.04	0.97	1.72	2.19
	identity	0.54	0.35	0.87	0.74	1.08	1.03	1.74	2.22

### Choosing a pool size

**Fig. 3:**
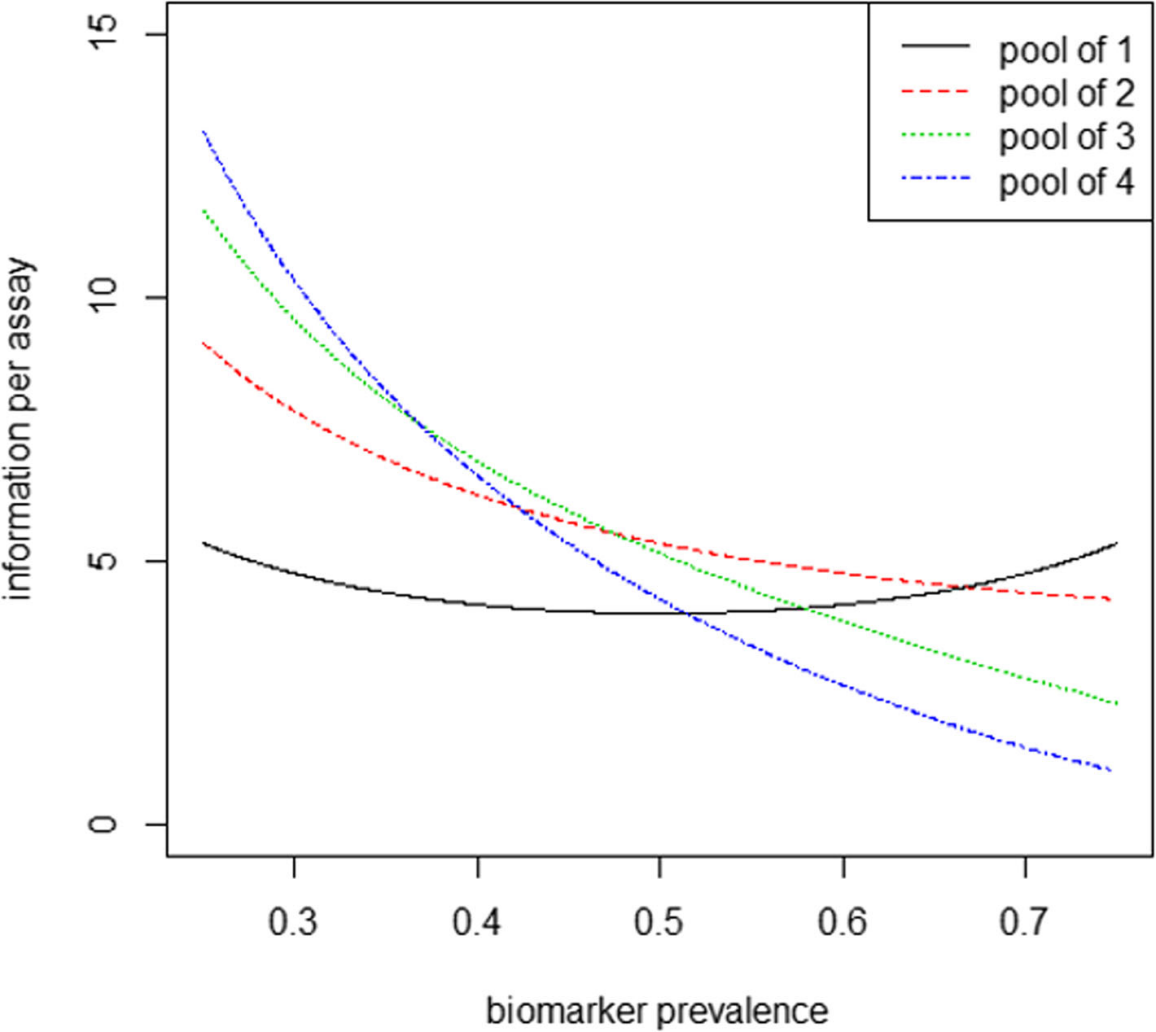
Fisher information in a pooled assay (of size 1 through 4) about biomarker prevalence as a function of the true prevalence

Figure 3 shows the per-assay Fisher information as a function of the true prevalence for four different pool sizes (1 through 4); the specific formula for any pool size is provided in the online supplement. Under the previously stated assumptions, Fig. 3 suggests that the optimal pool size among the four pool sizes with maximal cost-efficiency is 1 (i.e., no pooling) if the true prevalence of the biomarker is above 0.67 in the given stratum, 2 if the prevalence is between 0.48 and 0.66, 3 if the prevalence is between 0.37 and 0.47, and 4 or more if the prevalence is below 0.37.

## Discussion

To the best of our knowledge, this work is the first attempt to explore the use of GT in P-R biomarker studies. Our simulations and theoretical calculations have demonstrated that the GT design can be highly cost-efficient compared to both the standard design and the RS design, at least in some situations. Higher cost-efficiency translates into more efficient use of resources, which is desirable even as assay costs decline owing to technological advances.

We have assumed in the main text that assay error is negligible. While this assumption may be reasonable for some assays (such as the PCR-based assay employed in the E1900 trial), many assays have less-than-perfect accuracy, which should be incorporated in statistical estimation. In the online supplement, we provide estimation methods to account for possible misclassification and report an additional simulation study on the performance of GT designs when the assay is subject to misclassification. The additional simulation results indicate that GT designs generally achieve higher cost-efficiency than the standard and RS designs, consistent with the results in Tables 2 and 3.

An additional complication in the GT design is the well-known dilution effect, which may result in decreased sensitivity for pooled samples [33]. The magnitude of the dilution effect depends on assay specifics and may be expected to increase with pool size [34]. This issue has been considered by several authors in different contexts. For example, McMahan et al. [35] proposed a mechanistic modeling approach in which pool testing error rates are estimated from a rich set of low-level assay data; Hung and Swallow [36] and Zhang et al. [37] postulate that the pool testing error rates are known functions of the pool size and the number of diseased individuals in the pool. Further research is warranted on how to incorporate the dilution effect in GT designs of P-R biomarker studies.

We have assumed in this article that the biomarker is a binary indicator of the presence of some molecular alteration. If this is not the case, the relationship between a pooled assay result and individual assay results may become more complicated and more difficult to deal with in statistical estimation. For some continuous biomarkers, a pooled assay result may be plausibly assumed to be a (weighted) average of individual assay results, possibly with a random measurement error [38]. Novel statistical methods are needed to analyze GT designs with biomarkers that do not follow the pool-individual relationship assumed here.

Other areas of future research include development of statistical methods for GT designs with non-binary outcomes such as censored survival outcomes, which are commonly encountered in oncology trials, and optimization of GT designs for various combinations of outcomes and biomarkers.

## Conclusions

It has been demonstrated that group testing can substantially improve the cost-efficiency of prospective-retrospective biomarker studies. Further research is warranted to investigate the performance of the GT design in a wider range of real-world applications and to extend the statistical methods developed here to a greater variety of estimation problems.

## Supplementary Information

**Additional file 1.**

## Data Availability

We do not have the permission to distribute the E1900 trial data used in this article. However, the summary statistics needed to reproduce our simulation results are shown in Fig. 1.

## Abbreviations

GT: Group testing
HD: High-dose
P-R: Prospective-retrospective
RS: Random sampling
SD: Standard-dose
