# Supplementary material for: Group testing can improve the cost-efficiency of prospective-retrospective biomarker studies
Source: BMC Med Res Methodol. 2021 Mar 19;21:55. doi: 10.1186/s12874-021-01239-4 (PMC7977501; doi:10.1186/s12874-021-01239-4)
Supplement: Supplementary file 1 — Additional file 1. [file 12874_2021_1239_MOESM1_ESM.pdf]

Supplementary Material for “Group Testing Can Improve the Cost-Efficiency of Prospective-Retrospective Biomarker Studies”

Wei Zhang[1], Zhiwei Zhang[2,*], Julia Krushkal[2] and Aiyi Liu[3]

[1]LSC, Academy of Mathematics and Systems Science, Chinese Academy of Sciences, Beijing, China

[2]Biometric Research Program, Division of Cancer Treatment and Diagnosis, National Cancer Institute, National Institutes of Health, Bethesda, MD, USA

[3]Biostatistics and Bioinformatics Branch, *Eunice Kennedy Shriver* National Institute of Child Health and Human Development, National Institutes of Health, Bethesda, MD, USA

*zhiwei.zhang@nih.gov

Evaluating a Prognostic Biomarker

In general, a measure of association between X and Y, say $\delta g(p_1, p_0) = g(p_1) - g(p_0)$, can be estimated by substituting estimates of (p_1, p_0). If $\delta g(p_1, p_0)$ is the log-odds ratio, $\log[p_1(1 - p_0)/\{p_0(1 - p_1)\}]$, it can also be expressed in terms of (q_1, q_0) as $\log[q_1(1 - q_0)/\{q_0(1 - q_1)\}]$ (e.g., Agresti, 2013, Chapter 2), and thus can be estimated by substituting estimates of (q_1, q_0). For a different measure of association, $\delta g(p_1, p_0)$ is not a function of (q_1, q_0); however, estimates of (q_1, q_0) may still be useful for estimating (p_1, p_0) because, by Bayes' theorem,

$$
\begin{aligned}
p_1 &= \frac{\lambda q_1}{\lambda q_1 + (1-\lambda) q_0}, \\
p_0 &= \frac{\lambda (1 - q_1)}{\lambda (1 - q_1) + (1-\lambda)(1 - q_0)},
\end{aligned}
\tag{S.1}
$$

where $\lambda = \mathrm{P}(Y = 1)$.

The “full data”can be represented as (X_i, Y_i), $i = 1, \ldots, n$, where the subscript i denotes the ith subject in the trial. Under the standard design, the full data are fully observed, and it is straightforward to estimate p_x as a sample proportion:

$$
\widehat{p}_x^S = \frac{\sum_{i=1}^n I(X_i = x) Y_i}{\sum_{i=1}^n I(X_i = x)}, \qquad x = 0, 1,
$$

where $I(\cdot)$ is the indicator function and the superscript S denotes the standard design. The resulting estimate of $\delta g(p_1, p_0)$ is simply $\delta g(\widehat{p}_1^S, \widehat{p}_0^S)$.

Under the RS design, the X_i's are incompletely observed. Let $R_i = 1$ if X_i is observed; 0 otherwise. The RS design implies that

$$\mathrm{P}(R_i = 1 | X_i, Y_i) = \mathrm{P}(R_i = 1 | Y_i),$$

so X_i is missing at random in the sense of Rubin (1976). This further implies that

$$\mathrm{P}(X_i = 1 | Y_i = y, R_i = 1) = \mathrm{P}(X_i = 1 | Y_i = y) = q_y, \qquad y = 0, 1,$$

which motivates the following estimates:

$$\widehat{q}_y^{RS} = \frac{\sum_{i=1}^n I(R_i = 1, Y_i = y, X_i = 1)}{\sum_{i=1}^n I(R_i = 1, Y_i = y)}, \qquad y = 0, 1.$$

As noted earlier, if $\delta g(p_1, p_0)$ is the log-odds ratio, it can be estimated as $\delta g(\widehat{q}_1^{RS}, \widehat{q}_0^{RS})$. For other measures of association, we can invoke (S.1) and estimate (p_1, p_0) as

$$\begin{aligned}
\widehat{p}_1^{RS} &= \frac{\widehat{\lambda}\widehat{q}_1^{RS}}{\widehat{\lambda}\widehat{q}_1^{RS} + (1 - \widehat{\lambda})\widehat{q}_0^{RS}}, \\
\widehat{p}_0^{RS} &= \frac{\widehat{\lambda}(1 - \widehat{q}_1^{RS})}{\widehat{\lambda}(1 - \widehat{q}_1^{RS}) + (1 - \widehat{\lambda})(1 - \widehat{q}_0^{RS})},
\end{aligned}$$

where $\widehat{\lambda} = n^{-1} \sum_{i=1}^n Y_i$. The resulting estimate of $\delta g(p_1, p_0)$ is $\delta g(\widehat{p}_1^{RS}, \widehat{p}_0^{RS})$.

In the GT design, we allow pools in the same stratum to have different sizes for full generality. Suppose the subjects in the $Y = y$ stratum are randomly grouped into m_y pools of sizes k_{jy}, $j = 1, \ldots, m_y$. The marker status of the jth pool in the $Y = y$ stratum is given by $X_{jy}^* = \max_{1 \le i \le k_{jy}} X_{ijy}$, where X_{ijy} is the marker status of the ith subject in the same pool. It follows that

$$\mathrm{P}(X_{jy}^* = 1) = 1 - (1 - q_y)^{k_{jy}},$$

and the likelihood for q_y is

$$\prod_{j=1}^{m_y} \left\{1 - (1 - q_y)^{k_{jy}}\right\}^{X_{jy}^*} \left\{(1 - q_y)^{k_{jy}}\right\}^{1 - X_{jy}^*},$$

which can be maximized to estimate q_y. The resulting maximum likelihood estimates of (q_1, q_0) can be used to estimate $\delta g(p_1, p_0)$ in the same manner as in the RS design.

Evaluating a Predictive Biomarker

In general, the interaction coefficient β_{TX} can be estimated by substituting estimates of the p_{tx}'s into equation (2) in the main text. For the logit link,

$$\beta_{TX} = \log\left\{\frac{p_{11}(1-p_{10})(1-p_{01})p_{00}}{(1-p_{11})p_{10}p_{01}(1-p_{00})}\right\}$$

can be alternatively expressed as

$$\beta_{TX} = \log\left\{\frac{q_{11}(1-q_{10})(1-q_{01})q_{00}}{(1-q_{11})q_{10}q_{01}(1-q_{00})}\right\}; \tag{S.2}$$

see, for example, Liu et al. (2012, Supplementary Materials). Thus, in this case, β_{TX} can also be estimated by substituting estimates of the q_{ty}'s. For a different link function, β_{TX} is not a function of the q_{ty}'s but its estimation can be helped by estimation of the q_{ty}'s, as Bayes' theorem implies that

$$\begin{aligned} p_{t1} &= \frac{\lambda_t q_{t1}}{\lambda_t q_{t1} + (1-\lambda_t) q_{t0}}, \\ p_{t0} &= \frac{\lambda_t (1-q_{t1})}{\lambda_t (1-q_{t1}) + (1-\lambda_t)(1-q_{t0})}, \end{aligned} \tag{S.3}$$

where $\lambda_t = \mathrm{P}(Y=1|T=t)$, $t=0,1$.

In this setting, the full data can be represented as (X_i, T_i, Y_i), $i = 1, \ldots, n$, where the subscript i denotes the ith subject in the trial. Under the standard design, where all variables are fully observed, each p_{tx} can be estimated as a sample proportion:

$$\widehat{p}^{S}_{tx} = \frac{\sum_{i=1}^{n} I(T_i = t, X_i = x) Y_i}{\sum_{i=1}^{n} I(T_i = t, X_i = x)},$$

which can then be substituted into equation (2) to estimate β_{TX}.

Under the RS design, the X_i's are incompletely observed. Let $R_i = 1$ if X_i is observed; 0 otherwise. The RS design implies that

$$\mathrm{P}(R_i = 1|X_i, T_i, Y_i) = \mathrm{P}(R_i = 1|T_i, Y_i),$$

or equivalently,

$$\mathrm{P}(X_i = 1|T_i, Y_i, R_i = 1) = \mathrm{P}(X_i = 1|T_i, Y_i).$$

Therefore, we can estimate each q_{ty} with

$$\widehat{q}_{ty}^{RS} = \frac{\sum_{i=1}^{n} I(R_i = 1, T_i = t, Y_i = y, X_i = 1)}{\sum_{i=1}^{n} I(R_i = 1, T_i = t, Y_i = y)}.$$

These estimates can be substituted into equation (S.2) to estimate β_{TX} under the logit link. For other link functions, equation (S.3) suggests that each p_{tx} can be estimated as

$$\widehat{p}_{t1}^{RS} = \frac{\widehat{\lambda}_t \widehat{q}_{t1}^{RS}}{\widehat{\lambda}_t \widehat{q}_{t1}^{RS} + (1 - \widehat{\lambda}_t)\widehat{q}_{t0}^{RS}},$$

$$\widehat{p}_{t0}^{RS} = \frac{\widehat{\lambda}_t (1 - \widehat{q}_{t1}^{RS})}{\widehat{\lambda}_t (1 - \widehat{q}_{t1}^{RS}) + (1 - \widehat{\lambda}_t)(1 - \widehat{q}_{t0}^{RS})},$$

where $\widehat{\lambda}_t = \sum_{i=1}^{n} I(T_i = t)Y_i / \sum_{i=1}^{n} I(T_i = t)$, $t = 0, 1$. The $\widehat{p}_{tx}^{RS}$'s can be substituted into equation (2) to estimate β_{TX}.

For the GT design, suppose the subjects in the $(T = t, Y = y)$ stratum are randomly grouped into m_{ty} pools of sizes k_{jty}, $j = 1, \ldots, m_{ty}$. The marker status of the jth pool in the $(T = t, Y = y)$ stratum is given by $X_{jty}^{*} = \max_{1 \le i \le k_{jty}} X_{ijty}$, where X_{ijty} is the marker status of the ith subject in the same pool. It follows that

$$\mathrm{P}(X_{jty}^{*} = 1) = 1 - (1 - q_{ty})^{k_{jty}},$$

and the likelihood for q_{ty} is

$$\prod_{j=1}^{m_{ty}} \left\{1 - (1 - q_{ty})^{k_{jty}}\right\}^{X_{jty}^{*}} \left\{(1 - q_{ty})^{k_{jty}}\right\}^{1 - X_{jty}^{*}}.$$

Maximum likelihood estimates of the q_{ty}'s can be used to estimate β_{TX} in the same manner as in the RS design.

Choosing a Pool Size

When planning the retrospective part of a P-R biomarker study with GT, the relevant variance to minimize is the conditional variance of an estimator given observed data from the prospective part of the study. To fix ideas, consider a predictive biomarker study aiming to estimate β_{TX} for an arbitrary (but specified) link function g. Given $\mathcal{O} = \{(T_i, Y_i) : i = 1, \ldots, n\}$, the conditional variance of $\widehat{\beta}_{TX}^{GT}$ is a monotone function of the conditional variance

of $\widehat{\boldsymbol{q}}^{GT} = (\widehat{q}_{11}^{GT}, \widehat{q}_{10}^{GT}, \widehat{q}_{01}^{GT}, \widehat{q}_{00}^{GT})'$, the vector of maximum likelihood estimates of the q_{ty}'s. Specifically, $\mathrm{var}(\widehat{\beta}_{TX}^{GT}|\mathcal{O})$ decreases when $\mathrm{var}(\widehat{\boldsymbol{q}}^{GT}|\mathcal{O})$ becomes smaller in the sense of non-negative definiteness. Because $\mathrm{var}(\widehat{\boldsymbol{q}}^{GT}|\mathcal{O})$ is a diagonal matrix, $\mathrm{var}(\widehat{\beta}_{TX}^{GT}|\mathcal{O})$ is monotone in $\mathrm{var}(\widehat{q}_{ty}^{GT}|\mathcal{O})$ for each (t, y) pair. Now, consider a fixed (t, y) pair, and assume that the m_{ty} pools in the $(T = t, Y = y)$ stratum have the same size, say k. If m_{ty} is reasonably large, $\mathrm{var}(\widehat{q}_{ty}^{GT}|\mathcal{O})$ is approximately the inverse of the Fisher information about q_{ty} in $\{X_{jty}^*, j = 1, \dots, m_{ty}\}$, which is easily found to be $m_{ty} I_k(q_{ty})$, where

$$I_k(q_{ty}) = \frac{k^2(1 - q_{ty})^{2(k-1)}}{(1 - q_{ty})^k\{1 - (1 - q_{ty})^k\}} \tag{S.4}$$

is the Fisher information about q_{ty} in a single pooled assay result X_{jty}^*. If m_{ty} is fixed and n_{ty} is large enough, then the optimal value of k is the one that maximizes $I_k(q_{ty})$. Although this argument is made for a predictive biomarker, it can be applied to a prognostic biomarker with minor modifications.

Dealing with Misclassification

In the presence of possible misclassification, it is necessary to distinguish the true marker status Z from the measured marker status X based on a particular assay. Misclassification occurs when $X \neq Z$. To fix ideas, consider a predictive biomarker study aiming to estimate β_{TX} defined by equation (2) for some link function g. Note that the estimand has not changed despite possible misclassification, because X (not Z) is the biomarker being evaluated for potential adoption in clinical practice. Since X can be observed (fully or partially) in the standard and RS designs, no changes are required in the estimation methods described earlier for these designs. In the rest of this section, we will focus on developing appropriate estimation methods for the GT design.

We assume that misclassification is non-differential in the sense that

$$\mathrm{P}(X = 1|Z = z, T, Y) = \mathrm{P}(X = 1|Z = z) =: \phi_z, \qquad z = 0, 1. \tag{S.5}$$

In this notation, ϕ_1 and $1 - \phi_0$ are, respectively, the sensitivity and specificity of the assay. The values of (ϕ_0, ϕ_1) are assumed known from previous validation data. Any remaining

uncertainty about these values can be addressed in a sensitivity analysis. We further assume that there is no dilution effect in the sense that

$$\mathrm{P}(X^*_{jty} = 1|Z^*_{jty}) = Z^*_{jty}\phi_1 + (1 - Z^*_{jty})\phi_0, \tag{S.6}$$

where X^*_{jty} is a pooled assay result, $Z^*_{jty} = \max_{1\leq i\leq k_{jty}} Z_{ijty}$, Z_{ijty} is the true marker status of the ith subject in the jth pool of the $(T = t, Y = y)$ stratum, and k_{jty} is the size of the jth pool of the $(T = t, Y = y)$ stratum.

As before, the key to estimating β_{TX} in the GT design is the estimation of $q_{ty} = \mathrm{P}(X = 1|T = t, Y = y)$ for each (t, y) pair. Let $\gamma_{ty} = \mathrm{P}(Z = 1|T = t, Y = y)$; then it follows from assumption (S.5) and the law of total probability that

$$q_{ty} = \gamma_{ty}\phi_1 + (1 - \gamma_{ty})\phi_0. \tag{S.7}$$

Thus, an estimate of q_{ty} can be obtained by converting an estimate of γ_{ty}. To this end, we note that

$$\mathrm{P}(Z^*_{jty} = 0) = \mathrm{P}(Z_{ijty} = 0, i = 1, \ldots, k_{jty}) = (1 - \gamma_{ty})^{k_{jty}}$$

and assumption (S.6) then implies

$$\mathrm{P}(X^*_{jty} = 1) = \phi_0(1 - \gamma_{ty})^{k_{jty}} + \phi_1 \left\{1 - (1 - \gamma_{ty})^{k_{jty}}\right\}.$$

Therefore, the likelihood for γ_{ty} based on $\{X^*_{jty}, j = 1, \ldots, m_{ty}\}$ can be written as

$$\prod_{j=1}^{m_{ty}}\Bigg(\Big[\phi_0(1 - \gamma_{ty})^{k_{jty}} + \phi_1\big\{1 - (1 - \gamma_{ty})^{k_{jty}}\big\}\Big]^{X^*_{jty}} \times \Big[1 - \phi_0(1 - \gamma_{ty})^{k_{jty}} - \phi_1\big\{1 - (1 - \gamma_{ty})^{k_{jty}}\big\}\Big]^{1-X^*_{jty}}\Bigg).$$

Maximizing this likelihood yields the maximum likelihood estimate of γ_{ty}, which can be substituted into equation (S.7) to obtain the maximum likelihood estimate of q_{ty}. The resulting estimates of the q_{ty}'s can be used to estimate β_{TX} in the same manner as described earlier.

In what follows, we report a simulation study that incorporates assay error (i.e., misclassification). The simulation settings are the same as those in the "Methods" section, except that the assay is now imperfect with specificity $1 - \phi_0$ and sensitivity ϕ_1. We set

$1 - \phi_0 = \phi_1 \in \{0.90, 0.95, 0.99\}$. Table S1 below presents the simulation results in terms of relative efficiency and cost-efficiency for evaluating a predictive biomarker. When the sensitivity and specificity are both 0.99, the results are very similar to those in Table 3 (for a perfect assay). As the sensitivity and specificity go down, the relative efficiency and cost-efficiency of the GT designs decrease slightly. However, even when the sensitivity and specificity are 0.90, the GT designs remain competitive, with relative cost-efficiency 0.96-1.03 for GT-2 and 1.03-1.27 for GT-3. Thus, although misclassification appears to have an adverse effect on GT designs, such designs remain advantageous in cost-efficiency over the standard and RS designs.

Table S1. Simulation results for evaluating a predictive biomarker with misclassification in the setting of the E1900 trial. $1 - \phi_0$ and ϕ_1 are the specificity and sensitivity of the assay.

Biomarker	$1 - \phi_0$	Link for	Relative Efficiency				Relative Cost-Efficiency			
	$= \phi_1$	Interaction	RS-2	RS-3	GT-2	GT-3	RS-2	RS-3	GT-2	GT-3
FLD3-ITD	0.90	logit	0.54	0.36	0.50	0.37	1.09	1.07	1.01	1.11
		log	0.51	0.33	0.48	0.34	1.02	0.99	0.96	1.03
		identity	0.54	0.36	0.49	0.35	1.07	1.07	0.98	1.06
	0.95	logit	0.54	0.35	0.65	0.52	1.08	1.05	1.31	1.56
		log	0.50	0.32	0.64	0.51	1.01	0.96	1.27	1.53
		identity	0.53	0.35	0.64	0.51	1.07	1.05	1.28	1.54
	0.99	logit	0.55	0.35	0.80	0.67	1.10	1.06	1.60	2.02
		log	0.50	0.32	0.80	0.67	1.01	0.95	1.60	2.01
		identity	0.54	0.35	0.80	0.67	1.08	1.06	1.60	2.01
DNMT3A	0.90	logit	0.58	0.37	0.52	0.42	1.17	1.11	1.03	1.27
		log	0.53	0.34	0.50	0.39	1.07	1.03	1.00	1.18
		identity	0.56	0.36	0.50	0.40	1.11	1.08	1.00	1.21
	0.95	logit	0.57	0.37	0.67	0.57	1.14	1.12	1.34	1.71
		log	0.52	0.34	0.66	0.54	1.04	1.02	1.33	1.63
		identity	0.54	0.36	0.66	0.55	1.09	1.09	1.32	1.65
	0.99	logit	0.57	0.36	0.83	0.72	1.15	1.08	1.67	2.17
		log	0.53	0.33	0.82	0.69	1.07	1.00	1.64	2.08
		identity	0.55	0.35	0.82	0.70	1.11	1.06	1.65	2.11

References

Agresti A. *Categorical Data Analysis*, 3rd ed. 2013. John Wiley and Sons: Hoboken, NJ.

Liu A, Liu C, Zhang Z, Albert PS. Optimality of group testing in the presence of misclassi-

fication. *Biometrika* 2012; 99:245–251.

Rubin DB. Inference and missing data. *Biometrika* 1976; 63:581–592.
